# Effects of triazolodiazepine on the production of interleukin-6 from murine spleen cells and rabbit synovial cells *in vitro*

**DOI:** 10.1155/S0962935195000226

**Published:** 1995-03

**Authors:** Dian-Wen Ju, Qin-Yue Zheng, Xue-Tao Cao, Qing-Hua Zheng, Xiao-Jun Guan, Hong-Bin Wang

**Affiliations:** 1 Department of Immunology School of Basic Medicine Second Military Medical University Shanghai 200433 China; 2 Department of Pharmacology School of Pharmacy Second Military Medical University Shanghai 200433 China

## Abstract

Interleukin-6 (IL-6) is a multifunctional cytokine that regulates the immune response, acute phase anaphylactic reaction, and haematopoiesis. Lipopolysaccharide (6–24 μg/ml) significantly induced IL-6 release from murine spleen cells. In cultured rabbit synovial cells interleukin-1 (IL-1, 1–10 U/ml) induced IL-6 production in a concentration-dependent manner. Triazolodiazepine (Tri) is a hetrazepine platelet-activating factor antagonist. In this study we found that Tri (0.1–10 μmol/l) exerted strong inhibitory effects on LPS stimulated IL-6 production in murine spleen cells. Kinetic studies showed that the inhibition of IL-6 release was time-independent. In rabbit synovial cells Tri also reduced IL-6 release induced by IL-1 and tumour necrosis factor. Inhibition of cytokine production by Tri may partially explain its wide and strong anti-inflammatory effects.


Mediators of Inflammation 4, 130–132 (1995)

### Effects of triazolodiazepine on the production of interleukin-6 from murine spleen cells and rabbit synovial cells *in vitro*

**Dian-Wen Ju,[1,CA] Qin-Yue Zheng,[2] Xue-Tao Cao,[1] Qing-Hua Zheng,[1] Xiao-Jun Guan[1] and Hong-Bin Wang[2]**

[1]Department of Immunology, School of Basic Medicine, and [2]Department of Pharmacology, School of Pharmacy, Second Military Medical University, Shanghai 200433, Peoples' Republic of China

[CA] Corresponding Author

**INTERLEUKIN-6 (IL-6) is a multifunctional cytokine that regulates the immune response, acute phase anaphylactic reaction, and haematopoiesis. Lipopolysaccharide (6–24 μg/ml) significantly induced IL-6 release from murine spleen cells. In cultured rabbit synovial cells interleukin-1 (IL-1, 1–10 U/ml) induced IL-6 production in a concentration-dependent manner. Triazolodiazepine (Tri) is a hetrazepine platelet-activating factor antagonist. In this study we found that Tri (0.1–10 μmol/l) exerted strong inhibitory effects on LPS stimulated IL-6 production in murine spleen cells. Kinetic studies showed that the inhibition of IL-6 release was time-independent. In rabbit synovial cells Tri also reduced IL-6 release induced by IL-1 and tumour necrosis factor. Inhibition of cytokine production by Tri may partially explain its wide and strong anti-inflammatory effects.**

**Key words:** IL-6, Spleen cells, Synovial cells, Triazolodiazepine

### Introduction

Interleukin-6 (IL-6) is a multifunctional cytokine that regulates the immune response, acute phase anaphylactic reaction, and haematopoiesis.[1] It is known that IL-6 is produced by various cells.[2] Considering that IL-6 production may play a critical role in a number of disease, chronic inflammation, and lymphoid malignancies, relatively little is known about the effects of anti-inflammatory drugs on cytokine synthesis. It might therefore be worthwhile investigating the relationship between its inhibition and the mechanism of action of anti-inflammatory drugs. Triazolodiazepine (Tri) is a hetrazepine platelet-activating factor (PAF) antagonist. It exerts strong anti-inflammatory effects on many *in vivo* and *in vitro* models.[3] In the present study, the effects of Tri on the production of IL-6 from mouse splenocytes and cultured rabbit synovial cells were examined.

### Materials and Methods

*Animals and chemicals:* ICR mice weighing 16–24 g and New Zealand rabbits weighing 1.5–2.5 kg were purchased from the Zoological Experiment Center of the Second Military Medical University. RPMI-1640, MEM media and lipopolysaccharide (LPS, *Escherichia coli*, 055:B5) were purchased from Sigma Chemical Co., USA. Foetal calf serum (FCS) was supplied by the Department of Pathology, Second Military Medical University. Triazolodiazepine (Tri, WEB 2086) was a kind gift from Boehringer Ingelheim Co., Germany. [$^3$H]-TdR (814 TBq/mol) was obtained from Shanghai Institute of Nuclear Research, Chinese Academy of Sciences. Recombinant human tumour necrosis factor (TNF) was generously provided by Dr Y. Sohmura, Dainippon Pharmaceutical Co., Japan.

*Spleen cell preparation and IL-6 induction:* Splenocytes were prepared from spleens removed under sterile conditions from sacrificed ICR mice.[4] The cells were suspended in RPMI-1640 containing penicillin 100 U/ml, streptomycin 100 μg/ml, 2-mercaptoethanol 50 mmol/l and 10% FCS. The cells were cultured in sterile 24-well tissue culture plates (Corning, USA) at a density of $5 \times 10^6$ cells/ml/well with LPS alone or in combination with different concentrations of Tri. After 48 h incubation at 37°C in a 5% $CO_2$, humidified atmosphere, the supernatants were collected by centrifugation and stored −20°C until testing for IL-6 activity.

*IL-6 production from rabbit synovial cells in culture:* Rabbit synovial cells were cultured according to Ju.[5] Briefly, synovial membrane tissue was obtained aseptically from the knees of normal adult New Zealand rabbits. The membrane was cut into pieces and stuck to a culture flask with the inner side towards the flask wall. MEM medium supplemented with 10% FCS, penicillin (100 U/mol) and streptomy-

© 1995 Rapid Communications of Oxford Ltd cin (100 μg/ml) were added to the flask and cultivation was carried out at 37°C. When the cells had grown to confluence, they were trypsinized and seeded into a 24-well plate. Twenty-four hours later, the compounds to be tested were added to the cell monolayer. After 24 h incubation the supernatants were collected by centrifugation and stored at −20°C for IL-6 assay.

*IL-6 bioassay:* To measure IL-6 activity in the supernatants, the murine IL-6 dependent hybridoma cell line 7TD1 was used.[6] Briefly, cells were cultured on culture flasks in RPMI-1640 with 10% FCS and recombinant human IL-6 (gifts from Dr Steven Gillis, Immunex Co., USA). 7TD1 cells were seeded into 96-well flat-bottomed microtitre plates (Nunclon, Denmark) at a density of 1000 cells/well. Supernatants to be tested were added to the cells and incubated for 3 days in a humidified atmosphere. Cells were pulsed with [$^3$H]-TdR 18500 Bq for the last 12 h and were collected using a cell harvester. The incorporation of [$^3$H]-TdR was measured using a FJ-2107 scintillation counter (Xi-an, China).

### Results

*IL-6 production induced by LPS:* The levels of IL-6 production were determined by measuring the radioactivity of [$^3$H]-TdR incorporation in 7TD1 cells. LPS at 6–12 μg/ml markedly induced IL-6 production from murine spleen cells (Fig. 1).

*Inhibition of Tri on IL-6 production from splenocytes:* Splenocytes were incubated with LPS and Tri for 48 h. The release of IL-6 induced by LPS (12 μg/ml) was shown to be significantly reduced by Tri at 0.1–10 μmol/l in a concentration-dependent manner as indicated in Fig. 2.

*Time course of IL-6 production:* Tri co-cultured with LPS stimulated splenocytes for 24, 36, 48, 60 and 72 h. The data in Fig. 3 shows that at all times tested,

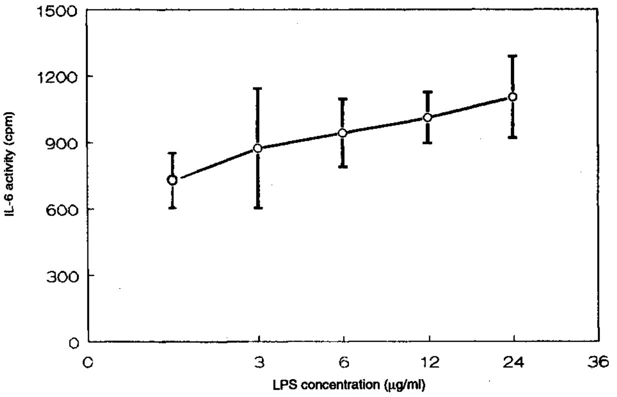

FIG. 1. Effect of lipopolysaccaride (LPS) on the production of interleukin-6 (IL-6) in mouse splenocytes. x ± S.D., $n = 6$, * $p < 0.05$, ** $p < 0.01$ *vs* control.

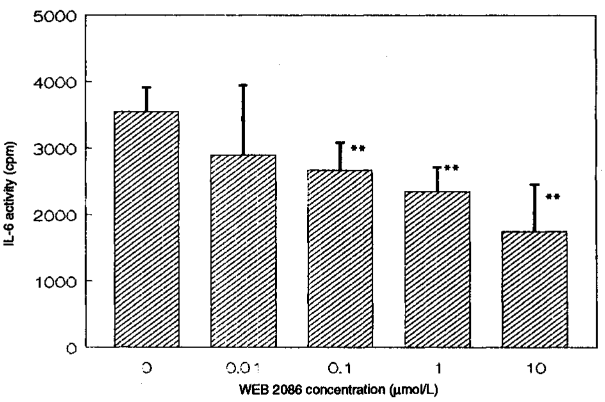

FIG. 2. Inhibitory effects of triazolodiazepine (Tri) on LPS (12 μg/ml) stimulated IL-6 production in murine splenocytes. x ± S.D., $n = 6$, * $p < 0.01$ *vs* control.

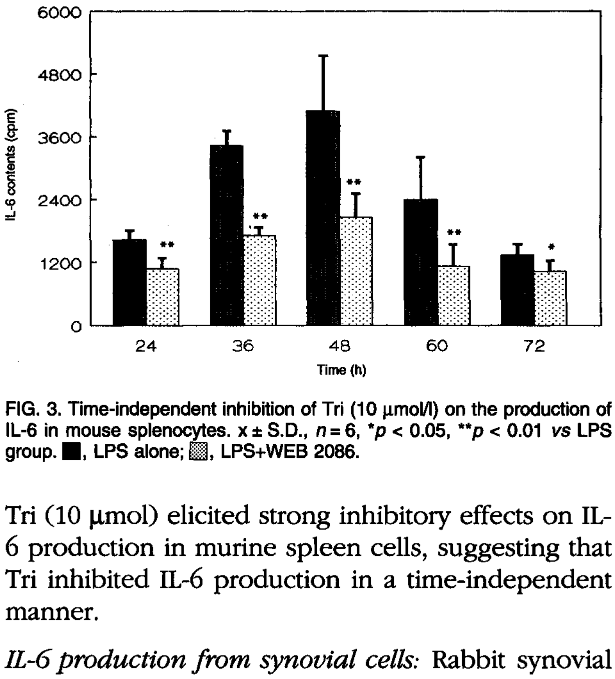

FIG. 3. Time-independent inhibition of Tri (10 μmol/l) on the production of IL-6 in mouse splenocytes. x ± S.D., $n = 6$, * $p < 0.05$, ** $p < 0.01$ *vs* LPS group. ■, LPS alone; ▒, LPS+WEB 2086.

Tri (10 μmol) elicited strong inhibitory effects on IL-6 production in murine spleen cells, suggesting that Tri inhibited IL-6 production in a time-independent manner.

*IL-6 production from synovial cells:* Rabbit synovial cells were stimulated with IL-1 and TNF for 24 h. The results demonstrated that IL-1 at 10 and 100 U/ml, TNF at 1 and 10 U/ml both significantly stimulated IL-6 release from synovial cells. The inducing effects seemed to be concentration-dependent (Table 1).

**Table 1.** Effects of tumour necrosis factor (TNF) and interleukin-1 (IL-1) on IL-6 production from cultured rabbit synovial cells. x ± S.D., $n = 6$, * $p < 0.05$, ** $p < 0.01$ *vs* control

| Drugs U/ml | IL-6 activity (cpm) |
|---|---|
| Control | 2081 ± 328 |
| IL-1 | |
| 1 | 2217 ± 256 |
| 10 | 2533 ± 374* |
| 100 | 3450 ± 476** |
| TNF | |
| 0.1 | 2398 ± 522 |
| 1 | 2870 ± 513** |
| 10 | 4253 ± 631** |

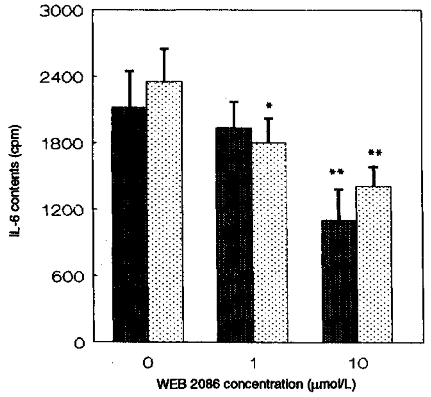

FIG. 4. Inhibitory effects of Tri on the production of IL-6 induced by IL-1 (100 U/ml) and TNF (10 U/ml) from rabbit synovial cells. x ± S.D., $n = 6$, *$p < 0.05$, **$p < 0.01$ *vs* control. ■, IL-1 (100 U/ml); ▒, TNF (10 U/ml).

*Reduction of IL-6 production from synovial cells by Tri:* IL-6 release challenged by IL-1 (100 U/ml) was markedly reduced by Tri (10 μmol/l). Tri also inhibited TNF (10 U/ml) induced IL-6 production at 1 and 10 μmol/l (Fig. 4).

### Discussion

IL-6 is known to have multiple actions on the growth, differentiation and function of lymphoid and non-lymphoid cells. It has recently been suggested that this cytokine plays a role in the pathogenesis of autoimmune disorders, in which excessive production of IL-6 may lead to abnormal B-cell differentiation and antibody production.[7] This study demonstrates that LPS significantly induced IL-6 production from murine spleen cells *in vitro*. Since splenocyte culture is a mixture of T-cells, B-cells and macrophages, the data here do not provide direct insight into the cellular source of IL-6. It is reported that macrophages, fibroblasts and endothelial cells are the main IL-6 producing cells,[8] but we also found that IL-1 and TNF stimulated IL-6 production in cultured rabbit synovial cells. IL-6 release from synovial cells in arthritic DBA/ij mice was reported by Sugita *et al.*[9] Elevation of IL-6 levels in sera and paws of autoimmune arthritic animals was also demonstrated.[10] In previous work it was found that IL-6 stimulated proliferation of synovial cells.[5] These data suggest that abnormal IL-6 production may be an important pathological parameter and a contributory factor in the proliferative lesions of arthritic disease.

Tri is a specific PAF antagonist. We found that Tri reduced the production of TNF from murine peritoneal macrophages, IL-1 and colony-stimulating factor from mouse splenocytes.[4,11] In this study we found that Tri exerted strong inhibitory effects on IL-6 production from LPS stimulated murine spleen cells and cultured rabbit synovial cells. We speculate that PAF may play an essential role in the production of these cytokines. PAF is a major inflammatory mediator which may interact with other cytokines such as IL-1, IL-6, CSF and TNF, thereby resulting in an autocatalytic augmentation of the inflammatory response.[3,12–14] Tri, being able to exhibit strong inhibitory effects on the production of these mediators, may be of great use in anti-inflammatory therapy.

### References

1. Hirano T. Interleukin-6 and its relation to inflammation and disease. *Clin Immunol Immunopathol* 1992; **62**: s60–s65.
2. Theisen-Popp P, Pape H, Muller-Peddinghaus R. Interleukin-6 (IL-6) in adjuvant arthritis of rat and its pharmacological modulation. *Int J Immunopharmacol* 1992; **14**: 565–571.
3. Koltai M, Hosford D, Guinot P, Esamu A, Braquet P. Platelet activating factor (PAF), a review of its effects, antagonists and possible future clinical implications (part I). *Drugs* 1991; **42**: 9–29.
4. Ju DW, Zheng QY, Wang HB, Fang J. Inhibitory effects of triazolodiazepine on mouse splenocytes and peritoneal macrophages *in vitro*. *Acta Pharmacol Sin* 1994; **15**: 65–68.
5. Ju DW, Zheng QY, Wang HB, Fang J. Effects of leflunomide on cytokine-induced DNA synthesis of rabbit synovial cells in culture. *Acta Pharmacol Sin* 1994; **15**: 223–226.
6. Engelberts I, von Asmuth EJU, van der Linden CJ, Buurman WA. The interrelation between TNF, IL-6, and PAF secretion induced by LPS in an *in vivo* and *in vitro* murine model. *Lymphokine Cytokine Res* 1991; **10**: 127–131.
7. Hirano T, Matsuda T, Turner M, *et al.* Excessive production of interleukin 6/B cell stimulating factor-2 in rheumatoid arthritis. *Eur J Immunol* 1988; **18**: 1797–1801.
8. Aarden LA, De Droot ER, Schaap OL, Lansdorp PM. Production of hybridoma growth factor by human monocytes. *Eur J Immunol* 1987; **17**: 1411–1416.
9. Sugita T, Ueno M, Furukawa O, Murakami T, Takata I, Tosa T. Effect of a novel anti-rheumatic drug, TA-383, on type II collagen-induced arthritis—suppressive effect of TA-383 on interleukin 6 production. *Int J Immunopharmacol* 1993; **15**: 515–519.
10. Sugita T, Furukawa O, Ueno M, Murakami T, Takata I, Tosa T. Enhanced expression of interleukin 6 in rat and murine arthritis models. *Int J Immunopharmacol* 1992; **15**: 469–476.
11. Ju DW, Zheng QY, Wang HB, Wang XF, Fang J. Effect of platelet activating factor antagonist WEB 2086 on the production of TNF from murine peritoneal macrophages. *Acta Pharm Sin* 1993; **28**: 721–727.
12. Braquet P. PAF/cytokine auto-generated feed back networks in microvascular immune injury: consequences in shock, ischemia and graft rejection. *J Lipid Mediat* 1989; **1**: 75–79.
13. Rola Pleszczynski M, Thivierge M, Ganon N, Lacasse C, Stankova J. Differential regulation of cytokine and cytokine receptor genes by PAF, $LTB_4$ and $PGE_2$. *J Lipid Mediat* 1993; **6**: 175–181.
14. Thivierge M, Rola Pleszczynski M. Platelet-activating factor enhances interleukin-6 production by alveolar macrophages. *J Allergy Clin Immunol* 1992; **90**: 796–802.

**Received 8 November 1994; accepted in revised form 10 January 1995**
